# The intracellular and plasma membrane pools of phosphatidylinositol-4-monophosphate control megakaryocyte maturation and proplatelet formation

**DOI:** 10.1016/j.rpth.2023.100169

**Published:** 2023-04-26

**Authors:** Ana Bura, Sara Čabrijan, Ivana Bertović, Antonija Jurak Begonja

**Affiliations:** University of Rijeka, Department of Biotechnology, Rijeka, Croatia

**Keywords:** megakaryocytes, PI4P, platelets, Sac1

## Abstract

**Background:**

Megakaryocytes (MKs) develop from hematopoietic stem cells after stimulation by the cytokine thrombopoietin. During megakaryopoiesis, MKs enlarge, undergo the process of endomitosis, and develop intracellular membranes (demarcation membrane system, DMS). During DMS formation, there is active transport of proteins, lipids, and membranes from the Golgi apparatus to the DMS. The most important phosphoinositide that controls anterograde transport from the Golgi apparatus to the plasma membrane (PM) is phosphatidylinositol-4-monophosphate (PI4P), whose levels are controlled by suppressor of actin mutations 1–like protein (Sac1) phosphatase at the Golgi and endoplasmic reticulum.

**Objectives:**

Here we investigated the role of Sac1 and PI4P in megakaryopoiesis.

**Methods:**

We analyzed Sac1 and PI4P localization in primary MKs derived from fetal liver or bone marrow and in the DAMI cell line by immunofluorescence. The intracellular and PM pools of PI4P in primary MKs were modulated by expression of Sac1 constructs from retroviral vector and inhibition of PI4 kinase IIIα, respectively.

**Results:**

We showed that in primary mouse MKs, PI4P is mostly found in the Golgi apparatus and the PM in immature MKs, while in mature MKs, it is found in the cell periphery and at the PM. The exogenous expression of wild-type but not C389S mutant (catalytically dead) Sac1 results in the perinuclear retention of the Golgi apparatus resembling immature MKs, with decreased ability to form proplatelets. The pharmacologic inhibition of PI4P production specifically at the PM also resulted in a significant decrease in MKs that form proplatelets.

**Conclusion:**

These results indicate that both intracellular and PM pools of PI4P mediate MK maturation and proplatelet formation.

## Essentials

• Phosphatidylinositol-4-monophosphate (PI4P) controls anterograde transport from the Golgi.
• We studied PI4P in relation to Golgi apparatus in primary mouse megakaryocytes (MKs).
• In immature MKs, PI4P is enriched at the Golgi, but in mature MKs, it is enriched at the plasma membrane.
• Disrupting PI4P localization or its levels leads to smaller MKs that produce fewer proplatelets.

## 1 Introduction

Megakaryocytes (MKs) are the largest (50-100 μm in diameter) and one of the rarest (0.01%) cells in the bone marrow (BM) [1]. They differentiate from hematopoietic stem cells in response to thrombopoietin, a major cytokine that is produced by the liver and binds to its receptor c-Mpl, promoting the development and growth of MKs [1,2]. During megakaryopoiesis, MKs undergo the process of endomitosis, increase in size, pack proteins into α- and dense granules, and develop a system of membranes called the demarcation membrane system (DMS) that is a source of membranes for future platelets (PLTs) [3]. The DMS is composed of numerous cisternae and tubules that are continuous with the PM, and it requires high levels of proteins and lipids for its formation [1]. The formation of DMS starts in the perinuclear region near the Golgi apparatus [4]. It has been shown that vesicles from the trans-Golgi network (TGN) are localized close to the DMS and fuse with the DMS, while the blockage of Golgi trafficking increases the number of immature MKs [4]. In the later stages of DMS formation, the endoplasmic reticulum (ER) makes membrane contact sites with the DMS [4]. This suggests that the transport vesicles deliver the necessary proteins, lipids, and membranes for the formation of DMS. One of the most abundant phosphoinositides (PIs) at the Golgi apparatus and the ER is phosphatidylinositol-4-monophosphate (PI4P) [5]. It can be produced by 4 different phosphatidylinositol-4 kinases (PI4Ks) divided into 2 subfamilies: type II kinases (PI4KIIα and PI4KIIβ) and type III kinases (PI4KIIIα and PI4KIIIβ) [6]. By binding effector proteins, PI4P regulates anterograde vesicular transport from the TGN to the plasma membrane (PM) [[7], [8], [9]]. Although PI4P is mostly present at the Golgi in animal cells, it can be found at the PM as well [5].

Suppressor of actin mutations 1–like protein (Sac1) is a SAC1 domain-containing phosphatase [10] that mainly dephosphorylates PI4P at the Golgi apparatus and ER [5]. It is a transmembrane protein with a CX5R(T/S) catalytic motif [11] that localizes to both ER and Golgi, which is growth factor–dependent [12]. When the cells are stimulated with growth factors, Sac1 is mostly found at the ER, resulting in the accumulation of PI4P at the Golgi apparatus and stimulation of the anterograde transport from the Golgi to the PM. In growth factor–deprived conditions, Sac1 mostly shifts from the ER to the Golgi apparatus, where it dephosphorylates PI4P, resulting in a decrease in anterograde trafficking from the Golgi apparatus to the PM. Data from the human and mouse PLT proteome revealed that Sac1 is highly expressed in human (5800 copies) and mouse (10,500 copies) PLTs [13], suggesting high copy numbers in MKs as well. Considering the proximity of the Golgi apparatus to maturing DMS, the contribution of its transport vesicles in supplying growing membranes, and the PI4P that regulates anterograde Golgi trafficking, we studied the role of PI4P and Sac1 in primary mouse MKs.

Here we show that in the early stages of primary mouse MK maturation, PI4P is enriched at the Golgi apparatus, while in the later stages, it is found to be enriched at the PM. The exogenous expression of wild-type Sac1 results in smaller MKs that produce fewer proplatelets. Since the inhibition of PI4P production at the PM by PI4KIIIα also results in a decrease in proplatelet formation, we suggest that both the intracellular and PM pool of PI4P mediate MK maturation and proplatelet formation.

## 2 Methods

### 2.1 Antibodies

Primary antibodies were obtained from the following resources: polyclonal rabbit anti-Sac1 (ABIN949071) was obtained from antibodies-online, polyclonal rabbit anti-Sac1 (HPA069869) was obtained from Atlas Antibodies, monoclonal mouse anti-GM130 (610823) was obtained from BD Transduction Laboratories, monoclonal mouse anti-PI4P IgM (Z-P004) was obtained from Echelon Biosciences, monoclonal mouse anti-KDEL (sc-58774) was obtained from Santa Cruz Biotechnology, polyclonal rabbit anti-TGN46 (ab16059) was obtained from Abcam, monoclonal rat anti-GPIbβ (M050-1) was obtained from Emfret Analytics, rabbit anti-β1 tubulin was a kind gift by Dr Joe Italiano (Brigham & Women’s Hospital, Harvard Medical School, Boston), phalloidin (A12379) conjugated with Alexa Fluor (AF)-488 was obtained from Invitrogen, polyclonal rabbit anti-PI4KIIIα (4902S) and polyclonal rabbit anti-GFP (2555S) were obtained from Cell Signaling, monoclonal mouse anti-IgM (MAB1326, R&D Systems) was a kind gift from Dr Jelena Ban (Laboratory of Molecular Neurobiology, Department of Biotechnology, University of Rijeka), and monoclonal mouse anti-GAPDH (MAB374), polyclonal rabbit anti-IgG (12-370), and polyclonal mouse anti-IgG (12-371) were obtained from EMD Millipore. Secondary antibodies were obtained from the following sources: goat anti-mouse conjugated with Alexa Fluor (AF)-555 (A21429), goat anti-rabbit conjugated with Alexa Fluor (AF)-555 (A21429), and goat anti-mouse conjugated antibodies with Alexa Fluor (AF)-488 (A11029) from Life Technologies; goat anti-mouse conjugated with Alexa Fluor (AF)-568 (A21043), goat anti-rabbit conjugated with Alexa Fluor (AF)-488 (A11070), and goat anti-rat conjugated antibodies with Alexa Fluor (AF)-650 (SA5-10021) from Invitrogen; and goat anti-rabbit IgG HRP-linked (7074S) and goat anti-mouse IgG HRP-linked (7076S) antibodies from Cell Signaling.

### 2.2 DAMI and HEK293T cells

DAMI cells were grown in Roswell Park Memorial Institute 1640 medium supplemented with 10% fetal bovine serum (FBS, Pan Biotech) and 1% penicillin/streptomycin (Lonza). On day 0, DAMI cells were differentiated with 100 nM phorbol 12-myristate 13-acetate (PMA, P8139-1MG, Sigma Aldrich) and cultivated for 4, 8, 24, and 48 hours in full medium or for 4, 8, and 48 hours in medium lacking 10% FBS. For transfection, DAMI cells were seeded and differentiated with 100 nM PMA and after 24 hours transfected with pEGFP-Sac1, pEGFP-C389S-Sac1 (a kind gift of Dr Gerry Hammond, Department of Cell Biology, University of Pittsburgh School of Medicine), or pmCherry-PI4P-SidM (a kind gift of Dr Antonella De Matteis, Telethon Institute of Genetics and Medicine Pozzuoli; Department of Molecular Medicine and Medical Biotechnology University of Napoli Federico II) with Lipofectamine 3000 transfection reagent (Thermo Fischer). Twenty-four hours after transfection, the cells were fixed and stained for proteins of interest. HEK293T cells were grown in Dulbecco’s modified Eagle’s medium (Pan Biotech) supplemented with 10% FBS (Pan Biotech) and 1% penicillin/streptomycin (Lonza). Cells were seeded in full medium for 24 hours before transfection with the EGFP-Murine Stem Cell Virus (MSCV) constructs using Lipofectamine 3000 transfection reagent or before lysis for western blotting.

### 2.3 Mice

C57BL/6J mice were used for all experiments. Mice were handled following the European Communities Council Directive of November 24, 1986 (86/609/EEC), and with institutional and national guidelines. All experimental procedures were approved by the Ethics Committees of the Department of Biotechnology and Medical School of the University of Rijeka (003-08/15-01/34; 322-01/22-01/02) as well as the Ministry of Agriculture of the Republic of Croatia (UP/I-322-01/15-01/123).

### 2.4 Mouse BM and fetal liver isolation and MK production

Mouse BM from 8- to 12-week-old mice and fetal liver (FL) from 13.5 days postcoitum old embryos were used. Cell aggregates from the BM were dissociated by mechanical aspiration 10 times through a 21G × 1” needle, 10 times through a 23G × 1” needle, and 2 times through a 25G × 5/8” needle. Dissociated cells from the BM and FL were filtered through a 70-μm cell strainer (Falcon) and centrifuged for 5 minutes at 1000 rpm. The cells were then resuspended and cultured in Dulbecco’s modified Eagle’s medium (Pan Biotech) supplemented with 10% FBS (Pan Biotech) and 1% penicillin/streptomycin (Lonza). For MK production, thrombopoietin (2% of the culture-producing cytokine) was added. Cells were cultivated for different periods, as indicated in the figures and figure legends. MKs were enriched through bovine serum albumin (BSA; CP84.2, Sigma) gradient on days 2 and 4 (FL–derived MKs) or days 3 and 5 (BM-derived MKs) for further analysis.

### 2.5 Proplatelet formation assay

On day 4 after isolation of FL–derived MKs, a BSA gradient was performed, and 18 hours later, the number of MKs forming proplatelets was counted and shown as the percentage of MKs that form proplatelets over all MKs. The counting of MKs and proplatelets was performed using an epifluorescent microscope (Olympus) or a bright-field microscope (Zeiss) at 20× magnification. For counting the number of MKs that form proplatelets after the inhibition of PI4KIIIα, 100 nM GSK-A1 (SYN-1219-M005, Adipogen) was used.

### 2.6 Mouse PLT isolation

Mouse PLTs were isolated from mouse peripheral blood. Peripheral blood was drawn in 1/10 of the Aster-Jandl anticoagulant (85 mM Na citrate, 69 mM citric acid, 20 g/L glucose, pH 4.6). Blood was centrifuged for 8 minutes at 100 *g*. Platelet rich plasma was diluted 1:5 in washing buffer (140 mM NaCl, 5 mM KCl, 12 mM trisodium citrate, 10 mM glucose, 12.5 mM sucrose; pH = 6) and centrifuged for 6 minutes at 100 *g*. Platelet rich plasma supernatant was taken and centrifuged for 5 minutes at 1200 *g*. The pellet was resuspended in 1 mL of washing buffer and again centrifuged for 5 minutes at 1200 *g*. The pellet was resuspended in 200 μL of resuspension buffer (10 mM HEPES, 140 mM NaCl, 3 mM KCl, 0.5 mM MgCl_2_, 0.5 mM sodium hydrogen carbonate, 10 mM glucose; pH = 7.4). PLTs were then left to rest for 30 minutes at 37 °C before lysing for western blot assay.

### 2.7 Constructs, retroviral production, and MK transduction

Human Sac1 (shares a 95.06% homology with the mouse protein) from pEGFP-Sac1 (wild-type [WT]) and C389S-Sac1 (mutated) from pEGFP-C389S-Sac1 were amplified by polymerase chain reaction with primers Fwd 5′ TAGGCGCCGGAATTAGATCACCGGTATGGTGAGCAAGGGCGAG 3′ and Rev 5′ ATCGGCCGCCAGTGTGCTGGAATTCAGTCTATCTTTTCTTTCTGGACC 3′. Both constructs were subcloned into a retroviral MSCV vector expressing EGFP into AgeI and EcoRI sites. For cloning of both constructs, NEBuilder HiFi DNA Assembly DNA Master Mix (E2621L, NEB) was used. To produce retroviruses, HEK293T cells were seeded for 24 hours and then cotransfected with MSCV constructs (EGFP-MSCV, EGFP-Sac1-MSCV, or EGFP-C389S-Sac1-MSCV) and pCL-Eco packaging plasmid using the transfection reagent Lipofectamine 3000. After 24 hours, the medium was changed, and after 72 hours, the viral supernatants were collected. Viral supernatants were filtered through a 0.45-μm filter and stored at −80 °C. On day 2 of the FL culture, the cells were transduced by adding retroviral supernatants in the presence of 8 mg/mL of polybrene (H9268, Sigma Aldrich). The samples were centrifuged at 800 *g* for 30 minutes and incubated at 37 °C for 3 hours. After replacement with fresh media, MKs were cultured for an additional 2 days. On day 3, FL–derived MKs were separated via BSA gradient and analyzed for the ability to form proplatelets on day 4. The percentage of MKs that produce proplatelets was calculated from the number of EGFP+ MKs producing proplatelets over the number of all EGFP+ MKs.

### 2.8 Immunostaining

DAMI cells were grown directly on coverslips, while MKs were spun down on glass coverslips using the Hettich cytospin centrifuge Rotofix 32A. For Sac1 with Golgi or ER markers, the cells were fixed with 4% paraformaldehyde (PFA) in phosphate-buffered saline (PBS) for 15 minutes at RT, rinsed 3 times with PBS, and permeabilized and blocked with 1% goat serum(P30-1001, Carl Roth)/0.2% BSA/0.05% saponin (4185.1, Carl Roth) for 45 minutes. The cells were incubated with primary antibodies (diluted 1:100) overnight at 4 °C or for 1 hour at RT depending on the manufacturer’s instructions. The cells were rinsed 3 times with PBS and incubated with secondary antibodies (diluted 1:500) for 1 hour at RT. The cells were rinsed again 3 times with PBS, stained with 4’,6-diamidino-2-phenylindole (D9542, Sigma Aldrich) in PBS for 1 minute, and rinsed and mounted.

Immunostaining of intracellular pools of PI4P, as well as Golgi markers through MK development, was performed as previously described [14]. Briefly, all steps were performed at room temperature. Cells were fixed with 4% PFA in PBS for 15 minutes, rinsed with PBS containing 50 mM NH_4_Cl, and permeabilized with 0.01% digitonin in buffer A (20 mM Pipes [pH 6.8], 137 mM NaCl, 2.7 mM KCl) for 5 minutes. Cells were then rinsed with buffer A, blocked (5% goat serum in PBS with 50 mM NH_4_Cl) for 45 minutes, and labeled with primary antibodies (diluted 1:100). The cells were then washed with buffer A, stained with secondary antibodies (diluted 1:500), postfixed for 5 minutes in 2% PFA, and mounted.

The staining of the PM pool of PI4P was performed with the modified protocol as described previously [15]. Briefly, cells were fixed with 4% PFA in PBS with 0.2% glutaraldehyde (GA) for 15 minutes and rinsed with PBS containing 50 mM of NH_4_Cl. All subsequent steps were performed on ice with all prechilled solutions. Cells were blocked and permeabilized with 0.5% saponin (in buffer A containing 5% goat serum and 50 mM of NH_4_Cl) for 5 minutes and blocked with buffer A containing 5% goat serum and 50 mM of NH_4_Cl for 45 minutes. Then, the cells were labeled with primary antibodies diluted 1:100 (anti-PI4P) or 1:200 (anti-GPIbβ), washed with buffer A, stained with secondary antibodies (1:500), postfixed for 10 minutes in 2% PFA on ice and an additional 5 minutes at room temperature, and then mounted.

For the staining of proplatelets, cells were fixed with 4% PFA in PBS for 15 minutes, rinsed with PBS, and permeabilized with 0.01% Triton X-100 in PBS for 10 minutes. Cells were then blocked (5% goat serum in PBS) for 45 minutes, labeled with primary antibodies, and diluted 1:100 or with phalloidin 488 and anti-GPIbβ (1:200). The cells were then washed with PBS, stained with secondary antibodies (1:500), and mounted.

### 2.9 Epifluorescent and confocal microscopy

Epifluorescent images were obtained using an IX83 Olympus microscope equipped with a Hamamatsu Orca R2 CCD camera. The objective used was a PLAPON 60×/1.42 Oil (Olympus). The Pearson correlation coefficient and the area of the cell were measured in FIJI ImageJ [16] software.

Confocal images were obtained using an LSM880 confocal microscope (Carl Zeiss) equipped with an Argon-laser multiline (458/488/514 nm) and HeNe laser (543 nm and 633 nm). The objective used was a Plan-Apochromat 63×/1.40 oil DIC III (Carl Zeiss). For ultrastructure visualization, the airy scan mode was used. Total fluorescence intensities (TFI) and the area of cells were measured using ZEN black software (Carl Zeiss), quantified on maximum z-projections, and expressed over the area (size) of cells. For fluorescence intensity measurements, imaging conditions between different samples were kept constant. Fluorescence intensity was always expressed over the area (size) of cells. Colocalization studies were performed using ZEN black software (Carl Zeiss).

### 2.10 Western blot

Cells were lysed with 3× SDS STOP/β-mercaptoethanol (200 mM Tris, 6% SDS, 15% glycerol, 10 mg bromophenol blue [pH 6.7]). Lysates were denatured for 5 minutes at 95 °C and proteins were separated with sodium dodecyl sulfate-polyacrylamide gel electrophoresis on 10% polyacrylamide gel at 100 V for 1 hour in the Bio-Rad system for vertical electrophoresis. For protein size tracking, PageRuler Plus Prestained Protein Ladder was used (Thermo Scientific, 26619). Proteins from the gel were electrotransferred onto the nitrocellulose membrane (Santa Cruz Biotechnology) in the Bio-Rad system at 0.45 A for 90 minutes. Membranes were blocked for 45 minutes in BSA/Tris-buffered saline with Tween (3% BSA [Pan Biotech, P06-1391100]/Tris, NaCl, 0.1% Tween 20) and incubated with primary antibodies overnight at 4°C. Membranes were then washed 3 times for 10 minutes with Tris-buffered saline with Tween, and secondary antibodies were added. Protein bands were visualized with ECL Prime Western Blotting detection reagents (GE Healthcare Life Sciences, RPN2236), and the pictures were taken with a Bio-Rad ChemiDoc MP Imaging System.

### 2.11 Statistical analysis

All experiments were performed at least in triplicate. Data are represented as mean ± SEM. Data were analyzed by *t*-test or anova using Prism software (GraphPad). Differences were considered significant when *P* values were < .05 (∗*P* < .05; ∗∗ *P* < .01; ∗∗∗*P* < .001; ∗∗∗∗*P* < .0001; ns*P* > .5).

## 3 Results

### 3.1 Endogenous Sac1 localizes mostly in the perinuclear region in BM– and FL–derived MKs

**Figure 1:**
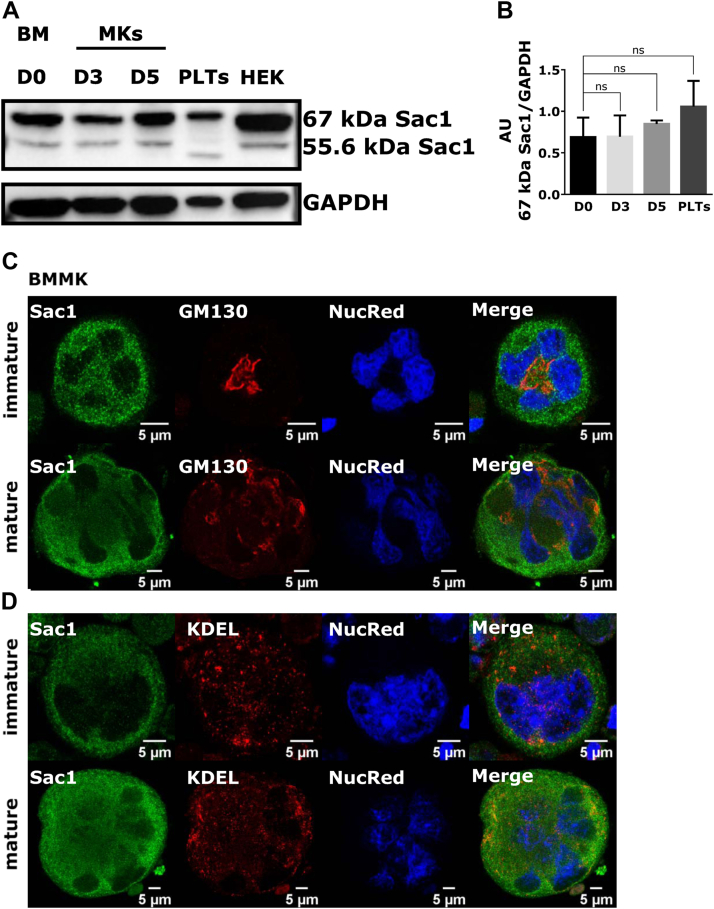
Sac1 is highly expressed and localized mostly at the ER in BM-derived MKs. MKs were isolated from the mouse BM, cultivated for 3 (immature) or 5 (mature) days, and enriched by a BSA gradient. PLTs were isolated from mouse peripheral blood, while HEK293T cells were seeded and cultured for 24 hours. (A) The cells were lysed, separated by SDS-PAGE, blotted onto a nitrocellulose membrane, and incubated with indicated antibodies. (B) Results in the graph are presented as means, error bars denote ±SEM from at least 3 independent experiments. ∗*P* < .05; ∗∗*P* < .01; ∗∗∗*P* < .001; ∗∗∗∗*P* < .0001; ns*P* > .05. (C, D) MKs were fixed and stained for (C) Sac1 and GM130 or (D) Sac1 and KDEL. Representative images display a single confocal optical section. The scale bar of the images is 5 μm. BM, bone marrow; BSA, bovine serum albumin; ER, endoplasmic reticulum; MK, megakaryocyte; n.s., nonsignificant; PAGE, polyacrylamide gel electrophoresis; PLT, platelet; SDS, sodium dodecyl sulfate.

**Figure 2:**
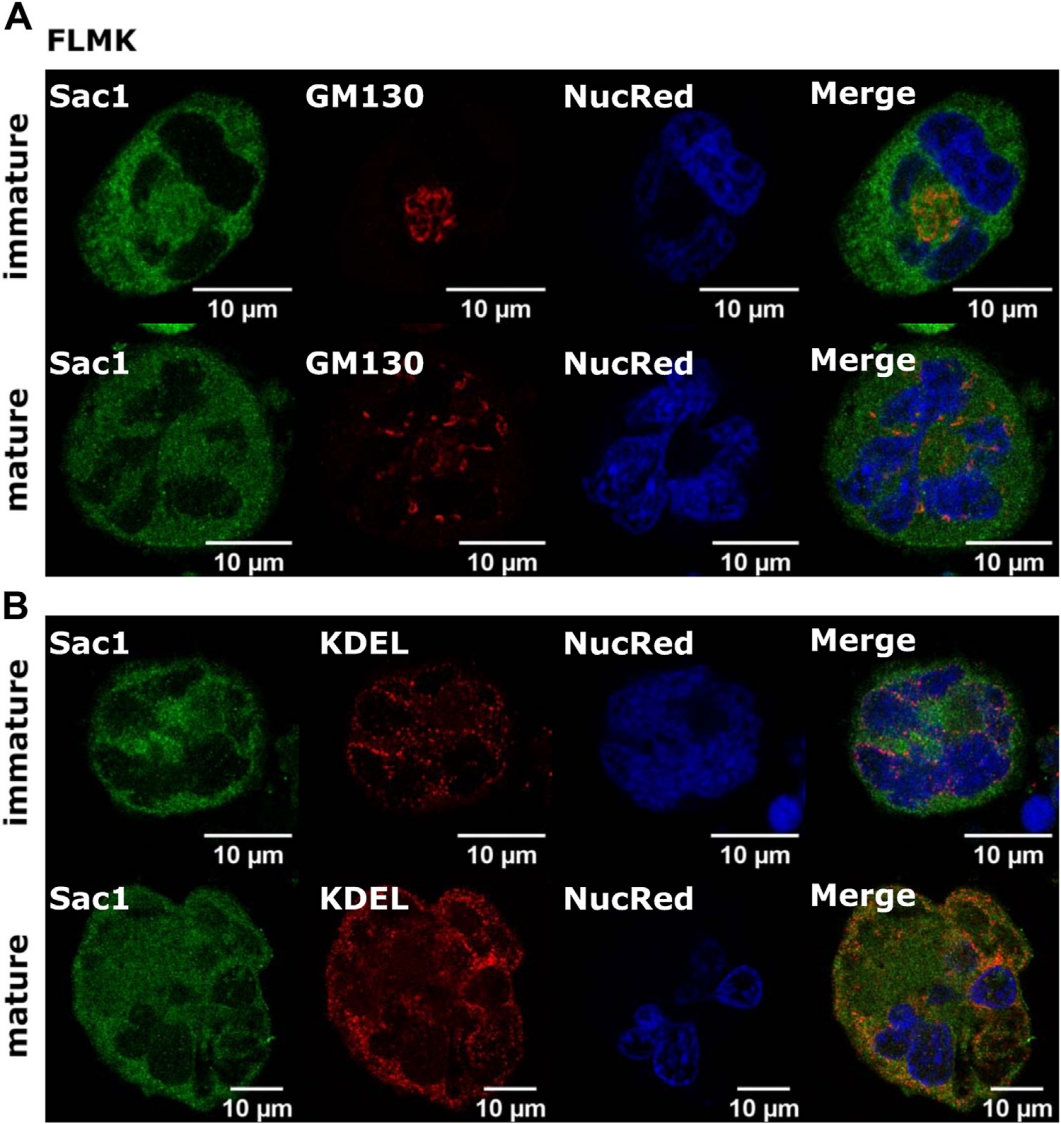
Sac1 localizes mostly at the ER in FL–derived MKs. MKs were isolated from the mouse FL, cultivated for 2 (immature) or 5 (mature) days, and enriched by a BSA gradient. MKs were fixed and stained for (A) Sac1 and GM130 or (B) Sac1 and KDEL. Representative images display a single confocal optical section. The scale bar of the images is 10 μm. BSA, bovine serum albumin; ER, endoplasmic reticulum; FL, fetal liver; MK, megakaryocyte.

First, we investigated the expression and localization of Sac1 in primary MKs. We cultivated MKs from mice BM for 3 or 5 days to produce immature or mature MKs, respectively (described in detail in reference [17]). Briefly, immature MKs are significantly smaller than mature MKs and they have a small nuclear/cytoplasmic ratio, while mature MKs enlarge 2- to 4-fold, have a large nuclear/cytoplasmic ratio, and increase the expression of GPIbβ. In BM-derived MKs, we could observe 2 Sac1 isoforms, a highly expressed 67 kDa isoform and an isoform of 55.6 kDa, with lower expression (Figure 1A). Further quantification of the highly expressed 67 kDa isoform showed that there was no change in expression levels during MK maturation and compared to mouse PLTs (Figure 1B, HEK293T as control cells). In immature MKs, Sac1 staining was predominantly vesicular, while in mature MKs, it was more dispersed (Figure 1C, D). In both immature and mature BM-derived MKs, Sac1 was following the shape and occasionally colocalizing with the ER marker KDEL (Figure 1D) and in the vicinity of the Golgi apparatus (Figure 1C). We observed the same phenotype in FL–derived MKs (Figure 2A, B). We confirmed the specificity of the used antibodies by staining for irrelevant IgG rabbit with anti-rabbit Alexa Fluor 488 secondary antibody (Supplementary Fig. S1A), irrelevant IgG mouse with anti-mouse Alexa Fluor 555 secondary antibody (Supplementary Fig. S1B), and secondary antibodies only (Supplementary Fig. S1C).

### 3.2 PI4P changes its localization from the Golgi apparatus toward the plasma membrane during MK maturation

**Figure 3:**
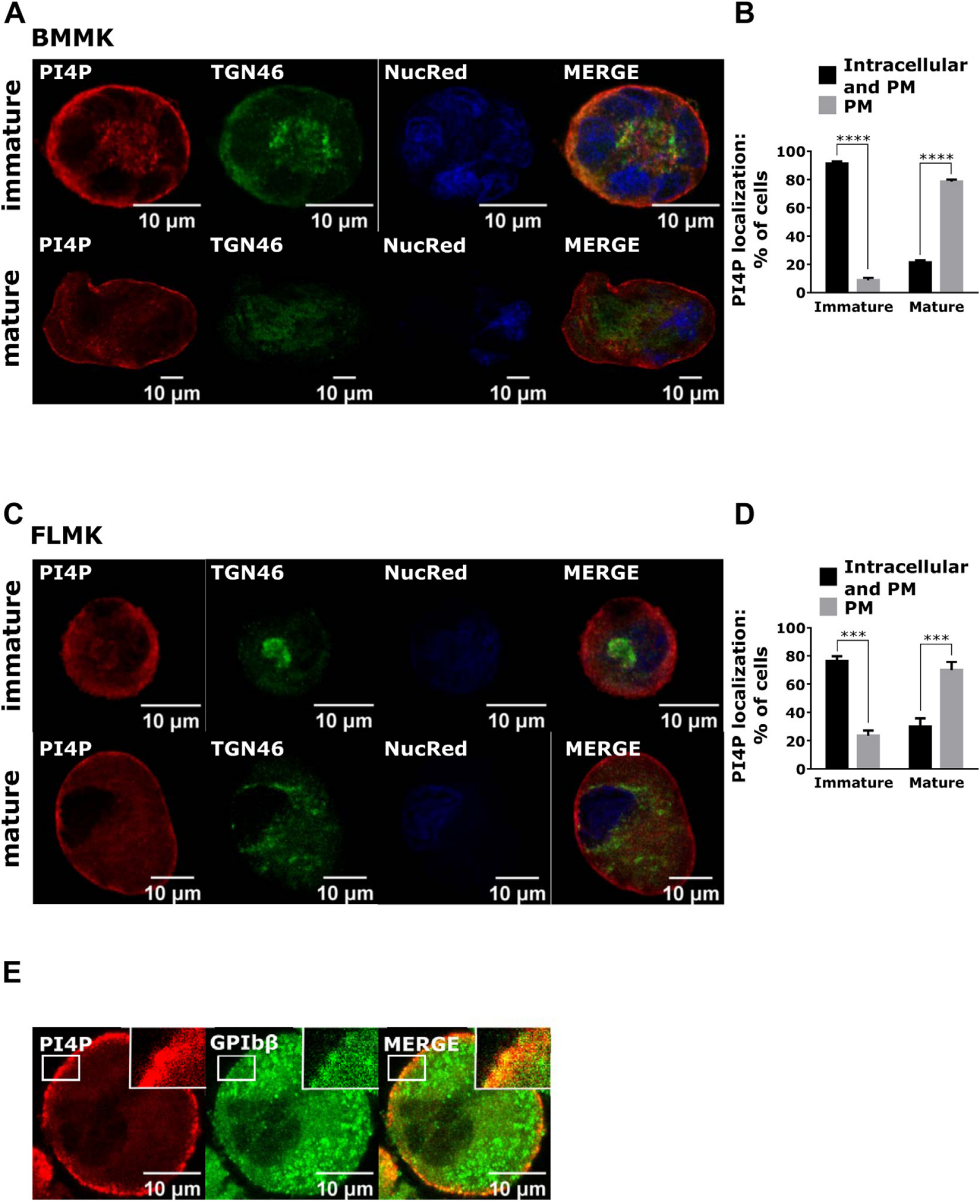
PI4P changes its localization from the Golgi apparatus toward the periphery of the cell and the PM in MKs. MKs were isolated from (A) the mouse BM or (C, E) mouse FL; cultivated for (A) 3 and 5 days, (C) 2 and 4 days, or (E) 4 days; and enriched by a BSA gradient. The cells were then fixed, stained for PI4P, and costained for TGN46 or GPIbβ. Representative images display a single confocal optical section. The scale bar of the images is 10 μm. (B, D) The number of MKs with different PI4P localization was counted. At least 30 cells per condition per experiment were counted. (E) MKs were fixed and stained for PI4P and GPIbβ. Results in the graphs are presented as means, and error bars denote ±SEM from at least 3 independent experiments. ∗*P* < .05; ∗∗*P* < .01; ∗∗∗*P* < .001; ∗∗∗∗*P* < .0001. BM, bone marrow; BSA, bovine serum albumin; FL, fetal liver; MK, megakaryocyte; n.s., nonsignificant; PI4P, phosphatidylinositol-4-monophosphate; PM, plasma membrane.

In other cells, PI4P is mostly localized on the Golgi apparatus but it can also be found at the PM [5]. In immature BM-derived MKs, PI4P indeed localized both at the Golgi apparatus, as shown by the colocalization with TGN46, and at the cell periphery and the PM (Figure 3A, upper panel). However, in mature MKs, it localized mostly at the periphery of the cell and PM (Figure 3A, lower panel). Quantification of the percentage of cells that show perinuclear and PM or PM-only staining of PI4P showed that approximately 90% of immature BM-derived MKs have perinuclear and PM PI4P pools, while approximately 80% of mature MKs had only PM PI4P pool (Figure 3B). This difference in PI4P localization was also observed in FL–derived MKs cultivated for 2 (immature) or 4 days (mature) [17]. In immature FL–derived MKs, PI4P localized perinuclearly at the Golgi apparatus and the PM (Figure 3C, upper panel), while in mature FL–derived MKs, it mostly localized at the periphery of the cell and the PM (Figure 3C, lower panel). Here the quantification revealed that approximately 80% of immature FL–derived MKs showed perinuclear and PM PI4P staining, while approximately 70% of mature FL–derived MKs showed only PM PI4P staining (Figure 3D). To confirm that PI4P localizes at the PM, we costained mature MKs with GPIbβ and observed PI4P and GPIbβ colocalization at the cell periphery (Figure 3E, inset). Previously, we tested the specificity of the anti-PI4P antibody in PLTs [15] and in MKs by staining for an irrelevant IgM mouse with anti-mouse Alexa Fluor 568 antibody (Supplementary Fig. S2A, B).

The change in PI4P localization could be due to the changes in the Golgi morphology itself. As shown in Supplementary Figure 3, the Golgi apparatus shown by the staining for trans-Golgi (TGN46) and cis-Golgi (GM130) in immature BM- and FL–derived MKs was localized perinuclearly, while in mature BM- and FL–derived MKs, it was dispersed in vesicular structures, as previously shown [4,18]. In BM-derived MKs, approximately 70% of immature MKs showed perinuclear localization of the Golgi apparatus, while approximately 80% of mature MKs had dispersed the Golgi into cytoplasmic vesicles (Supplementary Fig. S3B). Similar changes in the Golgi morphology happen in FL–derived MKs, where approximately 75% of immature MKs had perinuclear localization of the Golgi, while approximately 80% of mature MKs had Golgi structures dispersed into cytoplasmic vesicles (Supplementary Fig. S3D).

### 3.3 The expression of wild-type Sac1 negatively impacts megakaryocyte maturation

To determine the role of the intracellular pool of PI4P that is controlled by Sac1, we expressed WT and catalytically inactive C389S mutant Sac1 from retroviral vector in FL–derived MKs and assessed MK’s size, PI4P levels, and the ability of MKs to form proplatelets. The successful cloning of the Sac1 constructs in the retroviral vector was proved by transfecting HEK293T cells with the EGFP-MSCV, EGFP-Sac1-MSCV, and EGFP-Sac1-C389S-MSCV. The cells were then lysed and blotted with the anti-EGFP antibody (Supplementary Fig. S4A). The EGFP-MSCV bend was located at ∼28 kDa, while the Sac1 constructs coupled with EGFP were located at ∼95 kDa, which corresponds to the sum of the sizes of EGFP (28 kDa) and Sac1 (67 kDa).

To test the function of the cloned constructs before transducing primary MKs, we transfected DAMI cells. PI4P is mostly localized at the Golgi in untransfected DAMI cells (Supplementary Fig. S5, top panel). Interestingly, upon expression of WT Sac1, the levels of PI4P do not change (Supplementary Fig. S5B), but its localization changes. From its compact localization at the Golgi, PI4P disperses throughout the cell (Supplementary Fig. S5A) in approximately 80% of the cells (Supplementary Fig. S5C). In contrast, the expression of mutant Sac1 significantly increases PI4P levels (Supplementary Fig. S5B) with no change in its localization (Supplementary Fig. S5A, C). Since we could not observe a decrease in PI4P with the expression of WT-Sac1, we additionally tested if the antibody for PI4P is specific by expressing the P4M-SidM probe [19], which was shown to specifically bind to PI4P. As shown in Supplementary Figure 6, the P4M-SidM probe localizes at the Golgi where we could observe the staining of PI4P. This suggests that staining of PI4P is specific and obtained results with no change in PI4P levels could indicate that upon WT Sac1 expression, the cells try to compensate for the loss of PI4P by producing it ectopically via different pathways.

**Figure 4:**
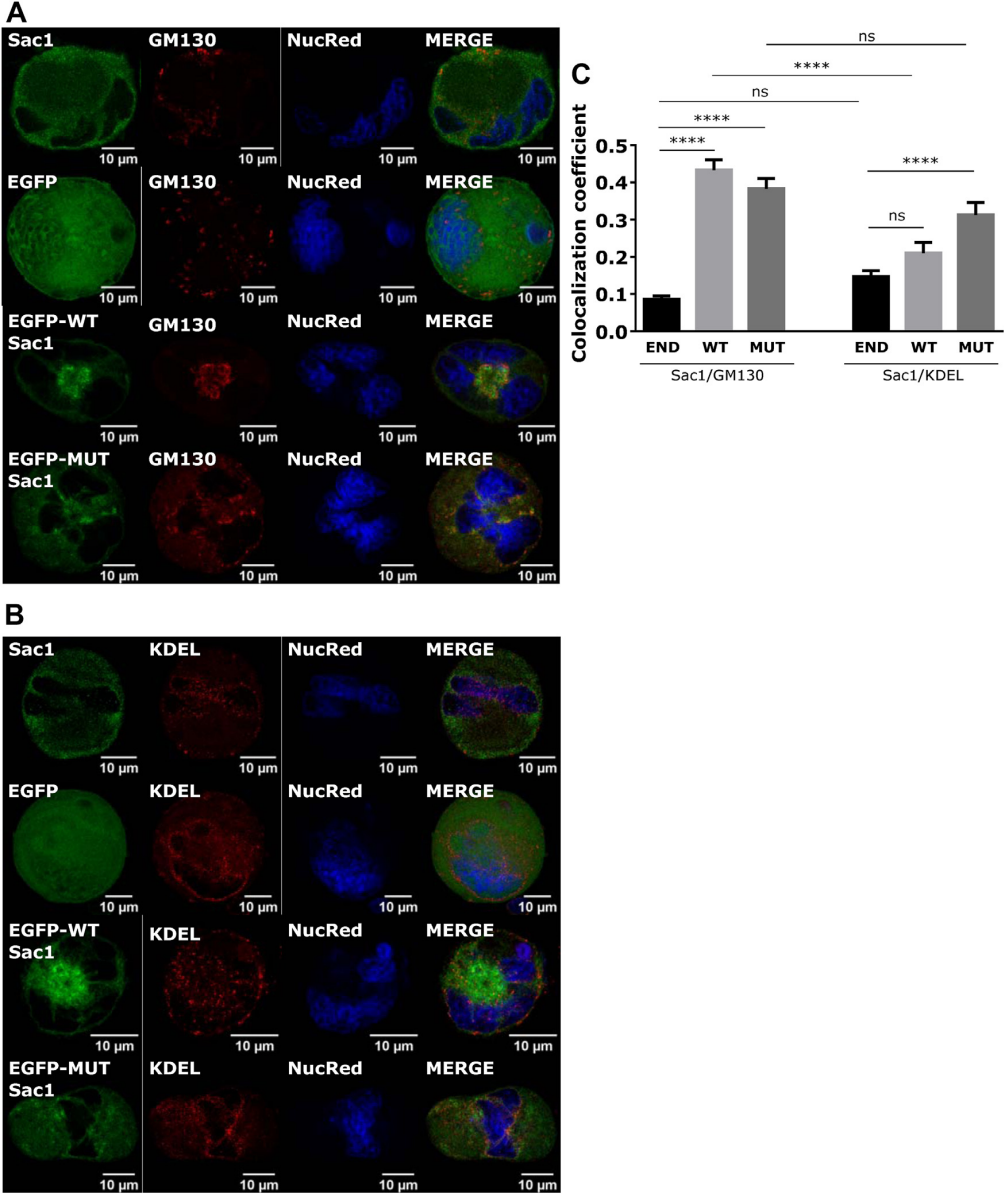
Exogenously expressed WT-Sac1 localizes mostly at the Golgi apparatus, while exogenously expressed mutant Sac1 localizes mostly at the ER. MKs were isolated from the mouse FL. On day 2 of the culture, they were infected with WT or MUT Sac1. On day 3 of the culture, they were enriched by a BSA gradient. On day 4 of the culture, they were fixed and stained for (A) Sac1 and GM130 or (E) Sac1 and KDEL. Representative images display a single confocal optical section. The scale bar of the images is 10 μm. (C) The colocalization coefficient of endogenous (END) or exogenous (WT or MUT) Sac1 with GM130 or KDEL was measured using ZEN black software. At least 15 cells per condition per experiment were measured. Results in the graphs are presented as means, and error bars denote ±SEM from 2 independent experiments. ∗*P* < .05; ∗∗*P* < .01; ∗∗∗*P* < .001; ∗∗∗∗*P* < .0001; ns*P* > .05. BSA, bovine serum albumin; ER, endoplasmic reticulum; FL, fetal liver; MK, megakaryocyte; MUT, mutant; n.s., nonsignificant; WT, wild-type.

**Figure 5:**
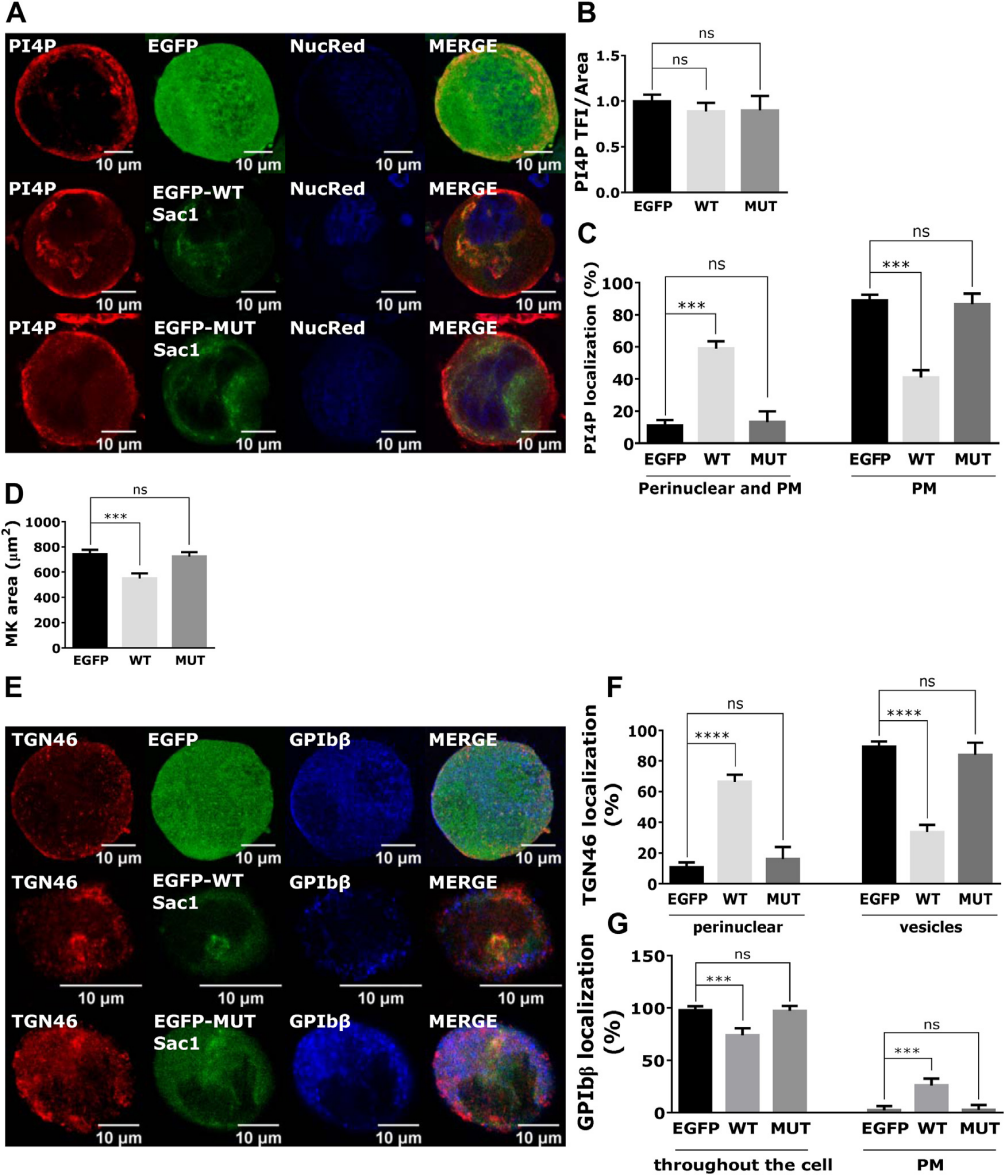
The expression of WT Sac1 negatively impacts MK maturation. MKs were isolated from the mouse FL. On day 2 of the culture, they were infected with WT or MUT Sac1. On day 3 of the culture, they were enriched by a BSA gradient. On day 4 of the culture, they were fixed and stained for (A) PI4P or (E) TGN46 and GPIbβ. (B) PI4P total fluorescence intensity, (C) intracellular and PM or PM-only PI4P localization, and (D) MK area were measured using ZEN black software. The percentage of MKs with (F) perinuclear or vesicular Golgi structures and (G) different GPIbβ localization was counted. The scale bar of the images is 10 μm. (B-D, F, and G) At least 15 cells per condition per experiment were measured. Representative images display a single confocal optical section. The scale bar of the images is (A, E) 10 μm. Results in the graphs are presented as means, and error bars denote ±SEM from at least 3 independent experiments. ∗*P* < .05; ∗∗ *P* < .01; ∗∗∗*P* < .001; ∗∗∗∗*P* < .0001; ns*P* > .05. BSA, bovine serum albumin; FL, fetal liver; MK, megakaryocyte; MUT, mutant; n.s., nonsignificant; PI4P, phosphatidylinositol-4-monophosphate; PM, plasma membrane; WT, wild-type.

Primary MKs were readily infected with the control EGFP as well as WT or mutant Sac1 constructs, with no significant difference in expression shown by western blot where infected cells were blotted for Sac1, and both endogenous and exogenous Sac1 can be detected (Supplementary Fig. S4B). The transduction rate between constructs was comparable, as shown by epifluorescent imaging (Supplementary Fig. S4C, D). We observed that WT Sac1 localizes mostly at the center of the cell in the perinuclear region, while mutant Sac1 is mostly scattered throughout cell (Figure 4A, B). We costained infected MKs with the marker of the Golgi apparatus (GM130) or the marker of the ER (KDEL) and measured the colocalization coefficient of endogenous Sac1, EGFP-WT-Sac1, and mutant Sac1 with the mentioned markers. Interestingly, expression of WT-Sac1, but not of mutant, changes the morphology of the Golgi apparatus, keeping it at the perinuclear region (further analyzed in Figure 5E, F). Quantification of the colocalization coefficient shows that endogenous Sac1 localizes more at the ER than at the Golgi apparatus in mature FL–derived MKs, although with no significant difference between the two (Figure 4C). However, the WT and mutant Sac1 localize significantly more at the Golgi apparatus than at endogenous Sac1 (Figure 4C). The colocalization of WT and mutant Sac1 was also higher with the ER marker than endogenous Sac1, and this was significant in MKs expressing mutant Sac1 (Figure 4C).

Interestingly, there was no significant difference in PI4P fluorescence intensity when WT or mutant Sac1 was expressed in MKs (Figure 5A, B). However, MKs expressing WT Sac1 showed PI4P staining more at the perinuclear region as well as at the PM, as opposed to control EGFP-expressing MKs that showed mostly PM staining (Figure 5A, C). This was consistent with their maturation state since MKs expressing WT Sac1 were significantly smaller than control MKs expressing EGFP or mutant Sac1 (Figure 5D). To further determine the maturation state of Golgi in MKs, we stained them with TGN46 together with the MK maturation marker GPIbβ, hypothesizing that if MKs are not fully mature, the Golgi apparatus would be localized more perinuclearly and less dispersed into cytoplasmic vesicles, while GPIbβ would be more at the PM and less throughout the cells representing DMS. MKs that were expressing WT Sac1 had a more perinuclear Golgi (∼70% of MKs), while the control MKs (90%) and the ones expressing mutant Sac1 (80%) had dispersed Golgi vesicles (Figure 5E, F). The difference in the GPIbβ localization was less dramatic; however, GPIbβ stained less throughout the cell in MKs expressing WT Sac1, also suggesting that they are less mature (Figure 5E, G). Furthermore, MK expressing WT Sac1 had slightly, although statistically insignificantly, increased GPIbβ TFI (Supplementary Fig. S4E). This is also consistent with the hypothesis that they are smaller and less mature, as shown previously [17].

### 3.4 PI4P is necessary for proplatelet formation

**Figure 6:**
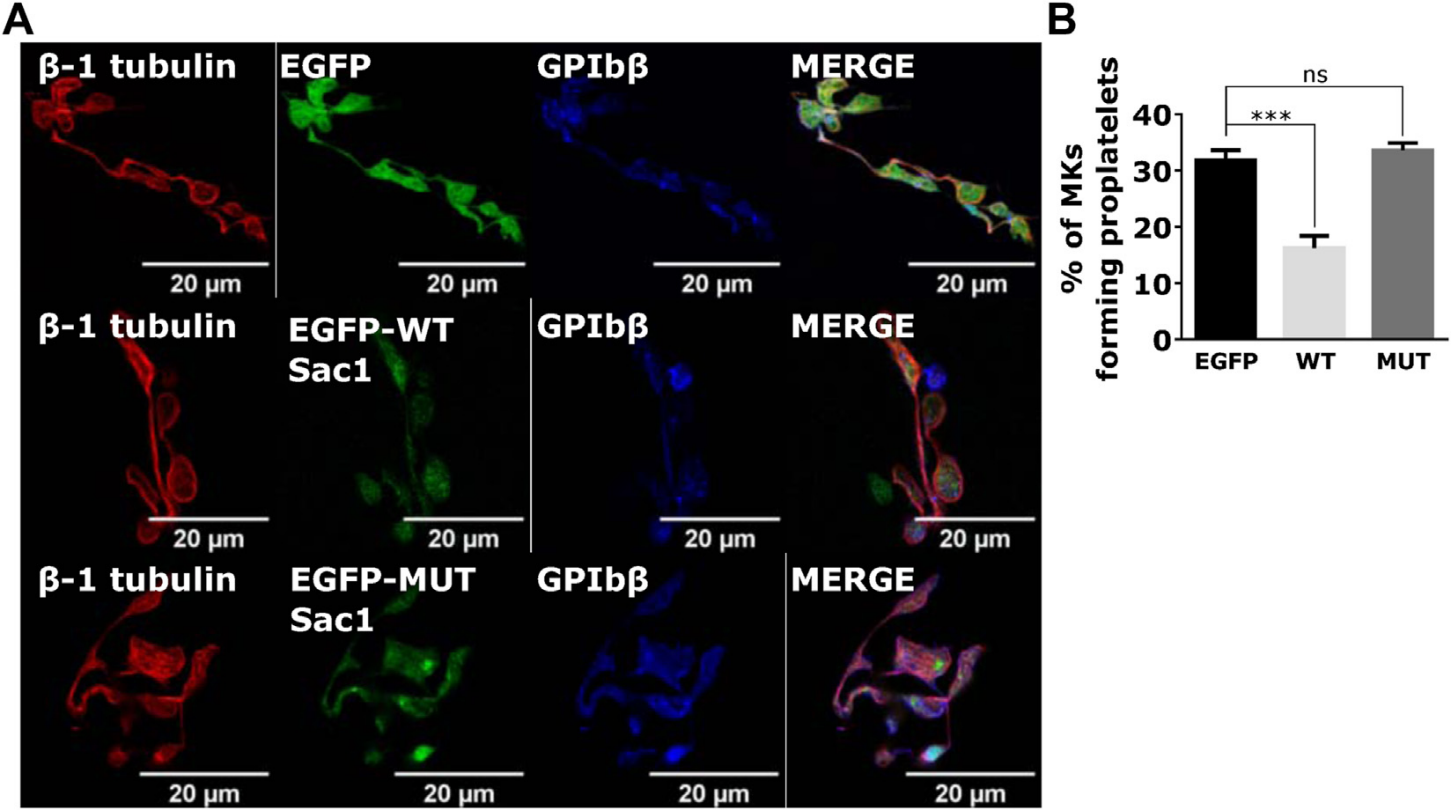
The expression of WT Sac1 decreases proplatelet formation. (A) MKs were isolated from the mouse FL. On day 2 of the culture, they were infected with EGFP only, EGFP-WT-Sac1, or EGFP-MUT-Sac1. On day 3 of the culture, they were enriched by a BSA gradient. On day 4 of the culture, proplatelets were fixed, stained for β1 tubulin, and costained for GPIbβ. Representative images display a single confocal optical section. The scale bar of the images is 20 μm. (B) The number of EGFP-expressing MKs with and without proplatelets was counted, and the percentage of MKs producing proplatelets was calculated. At least 5 fields of view per condition per experiment were counted. Results in the graph are presented as means, and error bars denote ± SEM from at least 3 independent experiments. ∗∗∗*P* < .001; ns*P* > .05. BSA, bovine serum albumin; FL, fetal liver; MK, megakaryocyte; MUT, mutant; n.s., nonsignificant; WT, wild-type.

We performed a proplatelet formation assay to determine the ability of Sac1-expressing MKs to produce proplatelets. MKs expressing WT Sac1 produced significantly fewer proplatelets than MKs expressing only EGFP or mutant Sac1 with no apparent change in proplatelet morphology (Figure 6A, B). These data suggest that the intracellular pool of PI4P controlled by the Sac1 phosphatase is important for proplatelet formation and that the perturbations in PI4P and/or Golgi localization do not allow full MK maturation and proplatelet formation.

**Figure 7:**
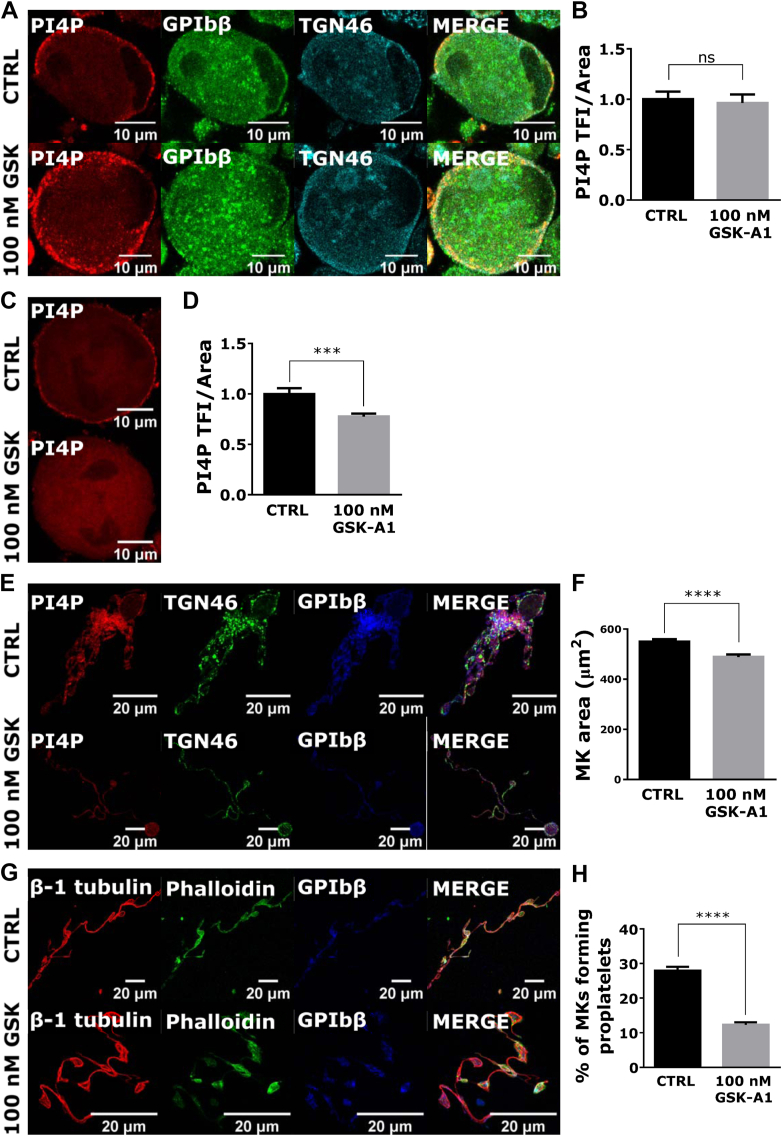
The inhibition of PI4P production at the PM significantly decreases MK ability to form proplatelets. MKs were isolated from the mouse FL. On day 3 of the culture, they were enriched by the BSA gradient and treated with 100 nM PI4KIIIα inhibitor GSK-A1. On day 4 of the culture, MKs were fixed, (A) stained for the intracellular pool of PI4P and costained for GPIbβ and TGN46, or (C) stained for the PM pool of PI4P. (B, D) PI4P total fluorescence intensity was measured using ZEN black software. On day 4 of the culture, (H) the percentage of MKs forming proplatelets was counted, and the proplatelets were fixed, stained for (E) PI4P and costained for TGN46 and GPIbβ or (G) β-1 tubulin, and costained with phalloidin and GPIbβ. (F) The MK diameter was measured using the AxioVision software. (B, D) At least 15 cells per condition per experiment were counted. (F) At least 50 cells per condition per experiment were counted. (H) At least 5 fields of view per condition per experiment were counted. The scale bars of the images are (A, C) 10 μm and (E, G) 20 μm. Representative images display a single confocal optical section. Results in the graphs are presented as means, and error bars denote ±SEM from at least 3 independent experiments. ∗*P* < .05; ∗∗*P* < .01; ∗∗∗*P* < .001; ∗∗∗∗*P* < .0001; ns*P* > .05. BSA, bovine serum albumin; FL, fetal liver; MK, megakaryocyte; n.s., nonsignificant; PI4P, phosphatidylinositol-4-monophosphate; PM, plasma membrane.

Since PI4P localizes more on the PM with MK maturation, we wanted to see if perturbation of the PM pool of PI4P would change the production of proplatelets. PI4KIIIα was shown to be the major source of PI4P at the PM in other cells [20] and it is highly expressed in both human (protein copy number: 1800) [13] and mouse (protein copy number: 1568) [21] PLTs. Here, we show that PI4KIIIα is highly expressed in mature BM- and FL–derived MKs, as well as mouse PLTs and HEK293T cells (Supplementary Fig. S7). Next, we pharmacologically inhibited PI4KIIIα with GSK-A1 (100 nM) and examined PI4P intensity and the ability of FL–derived MKs to produce proplatelets. For PI4P visualization, we used 2 staining methods: one that allows visualization of primarily the intracellular pool of PI4P (as used in all previous experiments) and one that allows visualization of only the PM pool of PI4P [14]. In MKs that were treated overnight with GSK-A1 (100 nM), there was no difference in the fluorescence intensity of the intracellular pool of PI4P, with no apparent changes in the localization of TGN46 or GPIbβ (Figure 7A, B). However, PI4KIIIα-inhibited MKs had significantly lower PI4P fluorescence intensity at the PM than control MKs (Figure 7C, D). Here, we do not show the TGN46 or GPIbβ staining since the PM staining protocol was not suitable for the detection of these proteins, which are found intracellularly. The change in PI4P levels is consistent with the expected localization of PI4KIIIα. Moreover, MKs treated with the PI4KIIIα inhibitor were somewhat smaller (Figure 7F) and produced significantly fewer proplatelets than the control MKs (Figure 7H). Proplatelets were clearly stained for PI4P along the shafts in controls, and staining was reduced upon PI4KIIIα inhibition. However, similar to the results obtained when expressing WT-Sac1, the morphology of the proplatelets produced by MK that were inhibited for PI4KIIIα did not change (Figure 7E, G). These data suggest that the PM pool of PI4P also contributes to proplatelet formation.

## 4 Discussion

Here we show that PI4P changes location during MK maturation. In young MKs, PI4P is mostly confined to the Golgi apparatus, while in mature MKs, it is mostly present at the PM. Both the intracellular and the PM pool of PI4P mediate MK maturation and proplatelet formation since the expression of WT-Sac1 and the inhibition of PI4KIIIα significantly reduce the number of MK-forming proplatelets. Although primarily found on the Golgi apparatus, PI4P also resides on the PM and ER in other cells [5], as well as late endosomes and lysosomes [19]. The role of PI4P at the PM is not clear. It was shown that it can serve as a substrate for the formation of phosphatidylinositol-4,5-bisphosphate [PI(4,5)P_2_], but it can also contribute to the polyanionic lipid pool that defines the inner leaflet of the PM [22]. A nonvesicular lipid transport driven by PI4P concentration gradient was recently described: lipid transfer protein domains at membrane contact sites transport lipid cargo from the ER to the Golgi or PM, and then, the same transfer domains traffic PI4P from membranes rich in this lipid (Golgi or PM) back to the ER where it is being hydrolyzed by Sac1. It is important to note that the knockout of Sac1 in mice is embryonically lethal, suggesting a housekeeping role of Sac1 [23].

In mature MKs in our experiments, it was not expected for PI4P to maintain its perinuclear localization since the Golgi apparatus itself undergoes morphology changes during megakaryopoiesis. In line with our observations, it has been previously shown that immature MKs have a condensed, perinuclear Golgi, while in mature MKs, the Golgi apparatus is widespread throughout the cell [4,18,[24], [25], [26]]. The Golgi apparatus may disperse to equip the DMS with needed proteins and lipids. It is possible that the dispersal of Golgi in mature MKs is preceded by the decrease in intracellular PI4P found in immature MKs. Rather, in mature MKs, PI4P is present in punctate structures in the periphery and on PM. A similar phenotype with fragmented Golgi and PI4P in punctate structures and peripheral membranes was observed when Sac1 was knocked down in other cells [23,27]. In our experiments, Sac1 is stably expressed at all stages of MK development, and although we did not observe different Sac1 localization with maturation, its appearance changes: in young MKs, it is observed in a punctate pattern that follows the ER, while in mature MKs, it is more dispersed (Figure 1C, D). Differential Sac1 activity could lead to intracellular PI4P hydrolysis; however, accumulation of PI4P at the PM could be due to PI4KIIIα production. The exogenous expression of WT-Sac1 in MKs results in a retention of the Golgi apparatus perinuclearly and its colocalization with TGN46, which subsequently leads to increased intracellular localization of PI4P (Figure 5). These results indicate that a balanced amount of functional Sac1 needs to be present in MKs. There is a clear correlation between exogenous Sac1 expression and PI4P/Golgi localization, leading to the formation of smaller MKs that produce significantly fewer proplatelets, while catalytically dead Sac1 had no effect. WT, but not catalytically dead Sac1, was shown to interact with COPI [28], and therefore, excess of WT-Sac1 molecules in MKs could bind additional COPI molecules and favor retrograde transport from the Golgi to the ER. In addition, an acute PI4P depletion was shown to diminish anterograde trafficking from the Golgi to PM and late endosomes [29]. Both of these scenarios could reduce the dispersion of the Golgi and consequently maturation of WT-Sac1–expressing MK.

The changes in Golgi morphology have been investigated in mice [4,18], rats [24,25], or guinea pigs [26] but not in human MKs. It would be interesting to see if the same applies to human MKs. MKs are not the only cells in which Golgi fragmentation has been observed. Small Golgi fragments have been found in neuronal dendrites that are separated from the perinuclear Golgi and serve as secretory organelles [30]. These neuronal Golgi fragments are called Golgi outposts (GOs) [31]. It has been shown that GOs are important for dendrite growth and branching because ablation of GOs reduces dendritic extension and retraction [32] and they can also interact with cytoskeletal remodeling and motor proteins [30]. Since GOs in neurons are important for dendrite growth, the Golgi fragments in MKs may have a similar role in the growth of the DMS and proplatelets. PI4P is the most important PI for anterograde Golgi trafficking, and it could serve as a driver of Golgi fragmentation.

Although we could not detect differences in overall PI4P levels in primary MKs or DAMI cells, the expression of WT Sac1 in DAMI cells dispersed PI4P, while in primary MKs, it caused accumulation of PI4P, indicating that diverse types of cells could differently regulate PI4P levels. For measuring PI4P levels, we used an established immunocytochemistry method [14] that was validated by expressing a PI4P-binding probe in the DAMI cell line (P4M-SidM). We tried to express different PI4P binding probes (FAPPb [9], OSBP [33]) in MKs; however, the transduction rate was very low (data not shown), which disabled the analysis of PI4P levels and localization with this method.

The lack of difference in PI4P fluorescence intensity levels accompanied by a change in localization of PI4P in WT-Sac1 expressing DAMI or primary MKs might be due to ectopic PI4P formation due to low intracellular PI4P levels. It was shown in HeLa cells that the inhibition of PI4KIIIα, a kinase that produces PI4P at the PM, leads to a decrease in PI4P levels at the PM with a simultaneous increase in intracellular PI4P [20]. PI4KIIIα is highly expressed in MKs, and its inhibition leads to a significant decrease in PI4P at the PM and a decrease in the formation of proplatelets, suggesting its role in the formation of PI4P at the PM. However, this does not exclude a role of other PI4 kinases in the process of MK maturation. While type III PI4Ks are cytosolic and shuffle between membranes of different cellular compartments or transiently associate with the PM [5], type II PI4Ks are mostly tethered at the Golgi and endosomal membranes [34]. Proteome data revealed that mouse PLTs express all 4 isoforms of PI4K; however, the most abundant isoform is PI4KIIIα [21] and only this isoform was found in human PLT proteome [13]. Our preliminary data indicate the expression of other PI4K isoforms also in mouse MKs, and thus could contribute to PI4P production and its different localization. Further studies are underway to decipher their role.

Several PI4P effector proteins have been shown to directly bind to PI4P in mammalian cells [35]. For some of the PI4P effector proteins, it has been shown to be highly expressed in mouse PLTs such as GGA1 (copy number: 1677), GGA3 (copy number: 342), and GOLPH3 (copy number: 1943) [21]. While GGAs are protein adaptors that localize at the TGN and mediate the transport from the Golgi to endosomes [36], GOLPH3 also localizes to the TGN via binding to PI4P but it is required for the exit of Golgi vesicles and their anterograde trafficking by inducing curvature of the Golgi membranes [37]. It is possible that perturbations in the PI4P levels or localization (eg, when WT-Sac1 is expressed) disable the Golgi recruitment of GOLPH3 to the TGN, which results in a defect of the Golgi anterograde trafficking. What are the exact PI4P effectors that mediate MK maturation remain to be elucidated.

In conclusion, we demonstrate that both the intracellular pool of PI4P that is controlled by the Sac1 phosphatase and the PM pool of PI4P that is controlled by the PI4KIIIα kinase are necessary for MK maturation and proplatelet formation. These results suggest that tight control of the levels and localization of PI4P is important for megakaryopoiesis.

## Supplementary material

Supplemental Figures Supplemental Figure 1. Antibodies for Sac1 and Golgi and ER markers are specific. MKs were isolated from the mouse BM cultivated for 3 (immature) or 5 (mature) days and enriched by a BSA gradient. The cells were then fixed and stained for **(A)** IgG rabbit with secondary Alexa Fluor anti-rabbit 488, **(B)** IgG mouse with secondary Alexa Fluor anti-mouse 555 or **(C)** secondary antibody Alexa Fluor anti-rabbit 488. Representative images display a single confocal optical section. The scale bar of the images is 5 μm. Supplemental Figure 2. The antibody for PI4P is specific. MKs were isolated from the mouse BM cultivated for 3 (immature) or 5 (mature) days and enriched by a BSA gradient. The cells were then fixed and stained for **(A)** PI4P or **(B)** IgM mouse with secondary Alexa Fluor anti-mouse 568. Representative images display a single confocal optical section. The scale bar of the images is 5 μm. Supplemental Figure 3. The Golgi apparatus disperses during MKs maturation. MKs were isolated from **(A)** the mouse BM or **(C)** the mouse FL, cultivated for **(A)** 3 and 5 days or **(C)** 2 and 4 days, and enriched by a BSA gradient. The cells were then fixed, stained for GM130 and co-stained for TGN46. Representative images display a single confocal optical section. The scale bar of the images is 10 μm. **(B** and **D)** The number of cells with different Golgi structures was counted. At least 30 cells per condition per experiment were counted. Results in the graphs are presented as means, error bars denote ± SEM from at least 3 independent experiments. ∗, p < 0.05; ∗∗, p < 0.01; ∗∗∗, p < 0.001; ∗∗∗∗ p < 0.0001; n.s., non-significant. Supplemental Figure 4. FL-derived MKs readily express WT and mutant Sac1. (A) HEK293T cells were seeded and after 24h transfected with the indicated constructs. The cells were then lysed, separated by SDS-PAGE, blotted onto a nitrocellulose membrane, and incubated with the anti-EGFP antibody. **(B)** For analysis of primary MKs, mouse FL cultures were infected with EGFP-, EGFP-WT or mutant Sac1-MSCV on day 2 of culture. Part of the cells was left uninfected (CTRL). On day 4 of culture, MKs were enriched by BSA gradient, lysed, separated by SDS-PAGE, blotted onto a nitrocellulose membrane, and incubated with anti-Sac1 antibody. **(C)** On day 4, the culture was imaged with an epifluorescent microscope, and **(D)** the percentage of MKs expressing different constructs was calculated. **(E)** The cells were then fixed and stained for GPIbβ (shown in Figure 5), and the GPIbβ TFI was measured. At least 15 cells per condition per experiment were counted. Representative images display a single confocal optical section. Results in the graphs are presented as means, error bars denote ± SEM from at least 3 independent experiments. ∗, p < 0.05; ∗∗, p < 0.01; ∗∗∗, p < 0.001; ∗∗∗∗ p < 0.0001; n.s., non-significant. Supplemental Figure 5. Exogenous expression of WT and mutant Sac1 changes PI4P levels and localization in DAMI cells. DAMI cells were differentiated with 100 nM PMA for 24h, transfected for WT or mutant Sac1 for an additional 24h, fixed, and stained for PI4P. The cells were analyzed by measuring **(B)** the mean fluorescence intensity of PI4P using FIJI ImageJ software and **(C)** the percentage of cells that have compact or dispersed PI4P staining. At least 15 cells per condition per experiment were measured. The fluorescence intensity graphs show the fluorescence intensity through the indicated arrows. Results in the graphs are presented as means, error bars denote ± SEM from at least 3 independent experiments. ∗, p < 0.05; ∗∗, p < 0.01; ∗∗∗, p < 0.001; ∗∗∗∗ p < 0.0001; n.s., non-significant. Supplemental Figure 6. P4M-SidM probe localizes at the TGN. DAMI cells were differentiated with 100 nM PMA for 24h and transfected with a P4M-SidM PI4P probe for an additional 24h. The cells were then fixed and stained for TGN46. The scale bar of the images is 20 μm. The fluorescence intensity graphs show the fluorescence intensity through the indicated arrows. Supplemental Figure 7. BM- and FL-derived MKs express PI4KIIIα. MKs were isolated from the mouse BM or FL, cultivated for 2 or 3 (immature), or 4 or 5 (mature) days, and enriched by a BSA gradient. PLTs were isolated from mouse peripheral blood, while HEK293T cells were seeded and cultured for 24h. The cells were lysed, separated by SDS-PAGE, blotted onto a nitrocellulose membrane, and incubated with indicated antibodies.
