# Supplementary material for: The intracellular and plasma membrane pools of phosphatidylinositol-4-monophosphate control megakaryocyte maturation and proplatelet formation
Source: Res Pract Thromb Haemost. 2023 Apr 26;7(4):100169. doi: 10.1016/j.rpth.2023.100169 (PMC10251075; doi:10.1016/j.rpth.2023.100169)

Supplemental Figure 1

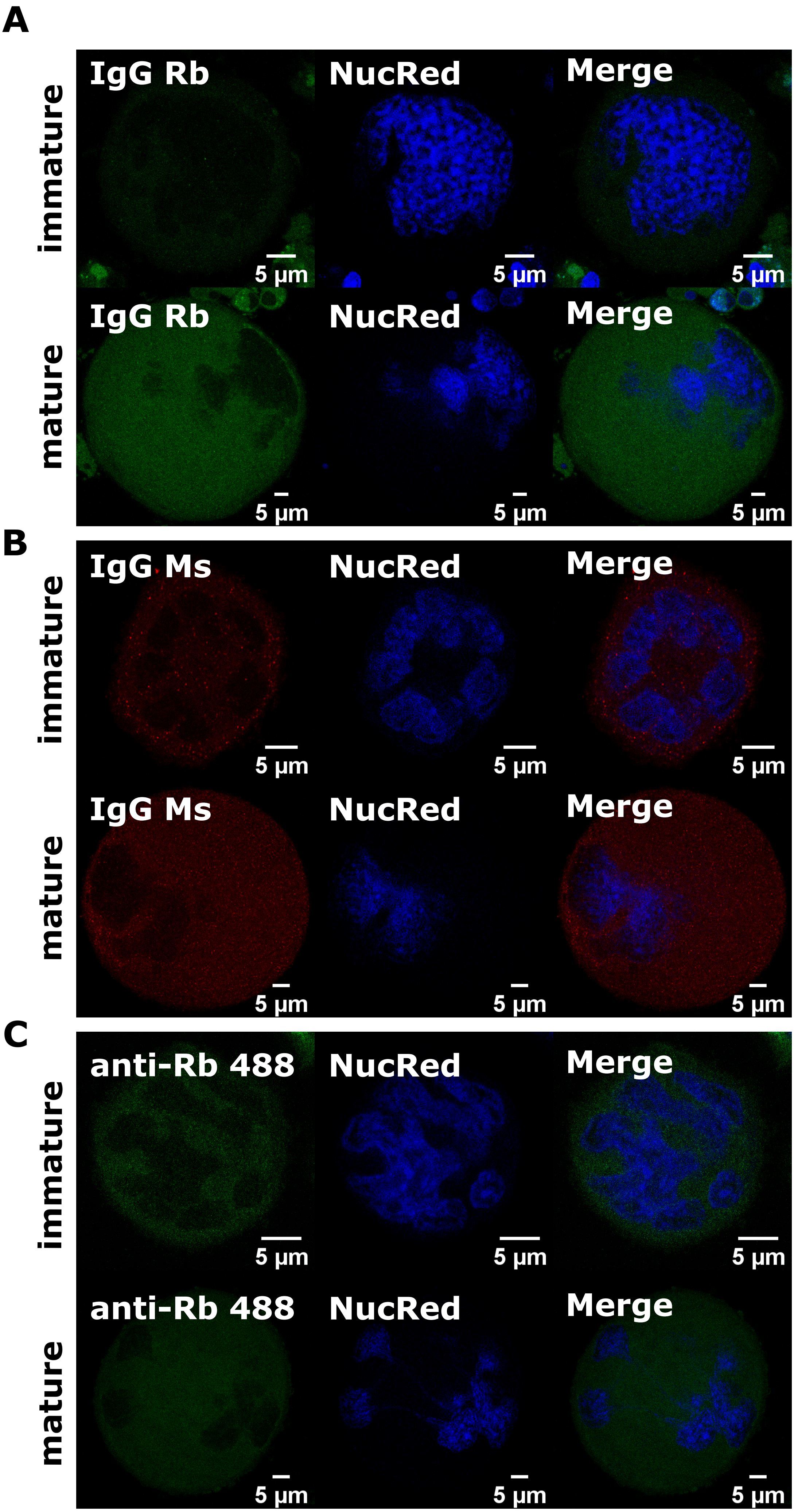

Supplemental Figure 2

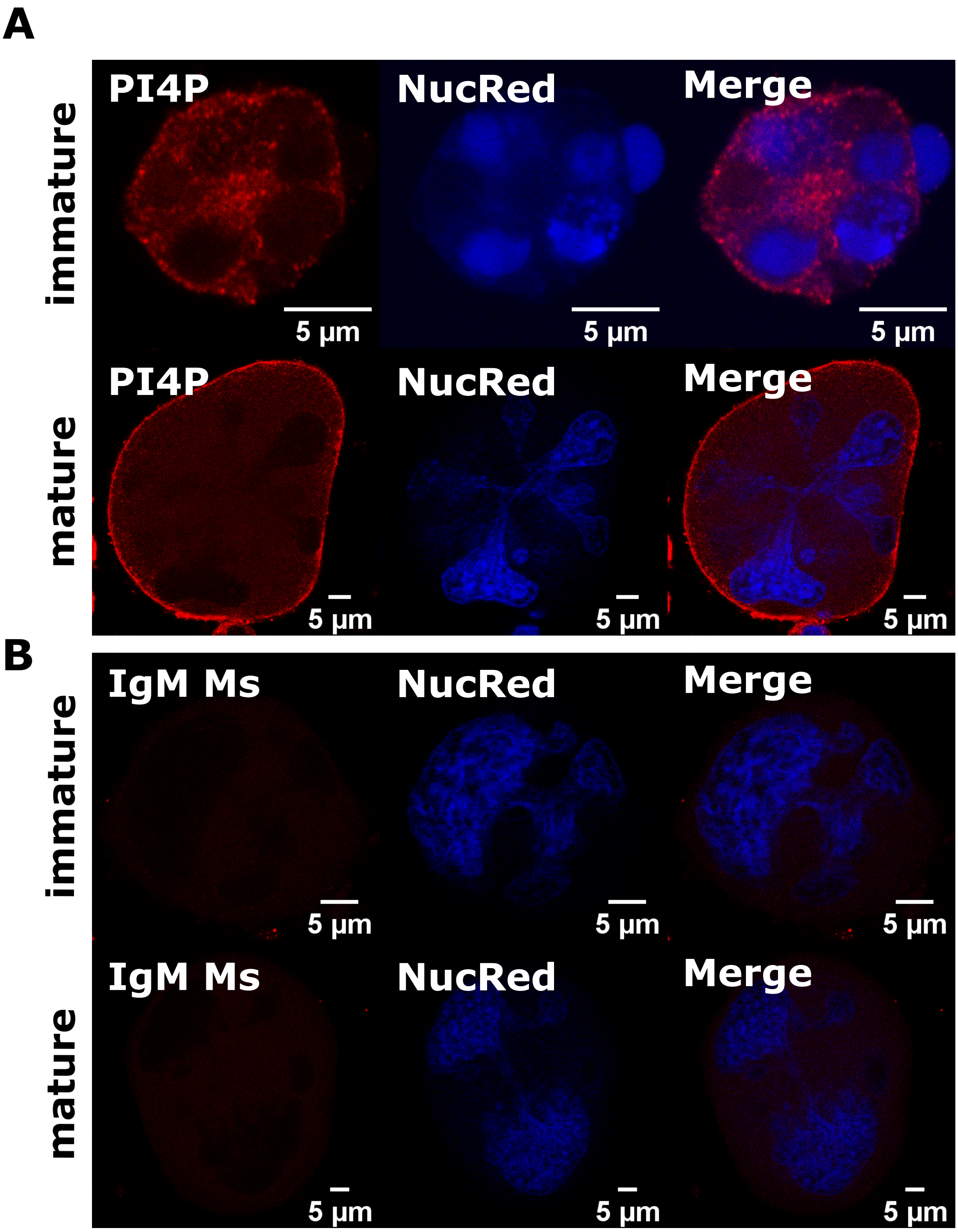

Supplemental Figure 3

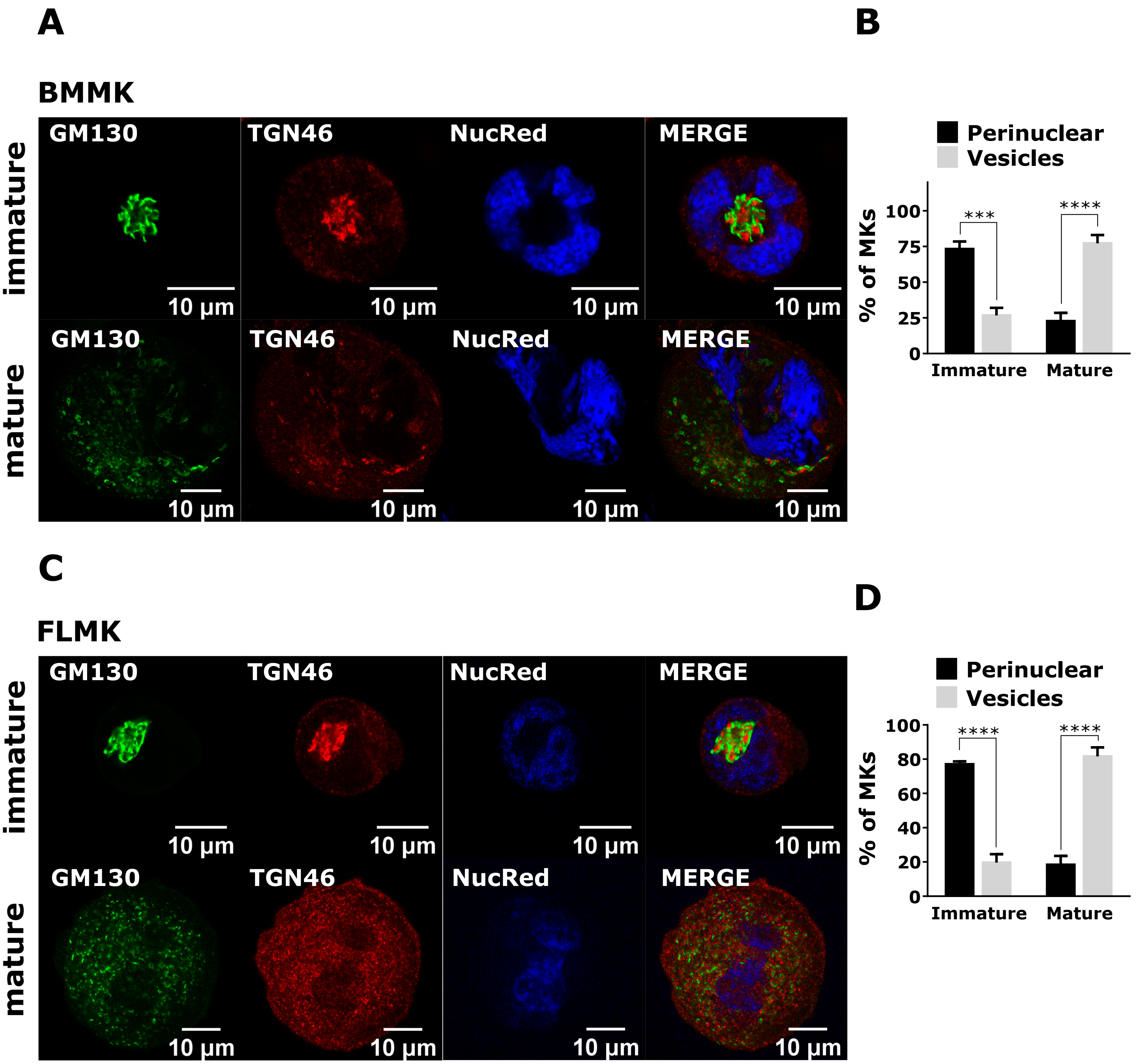

Supplemental Figure 4

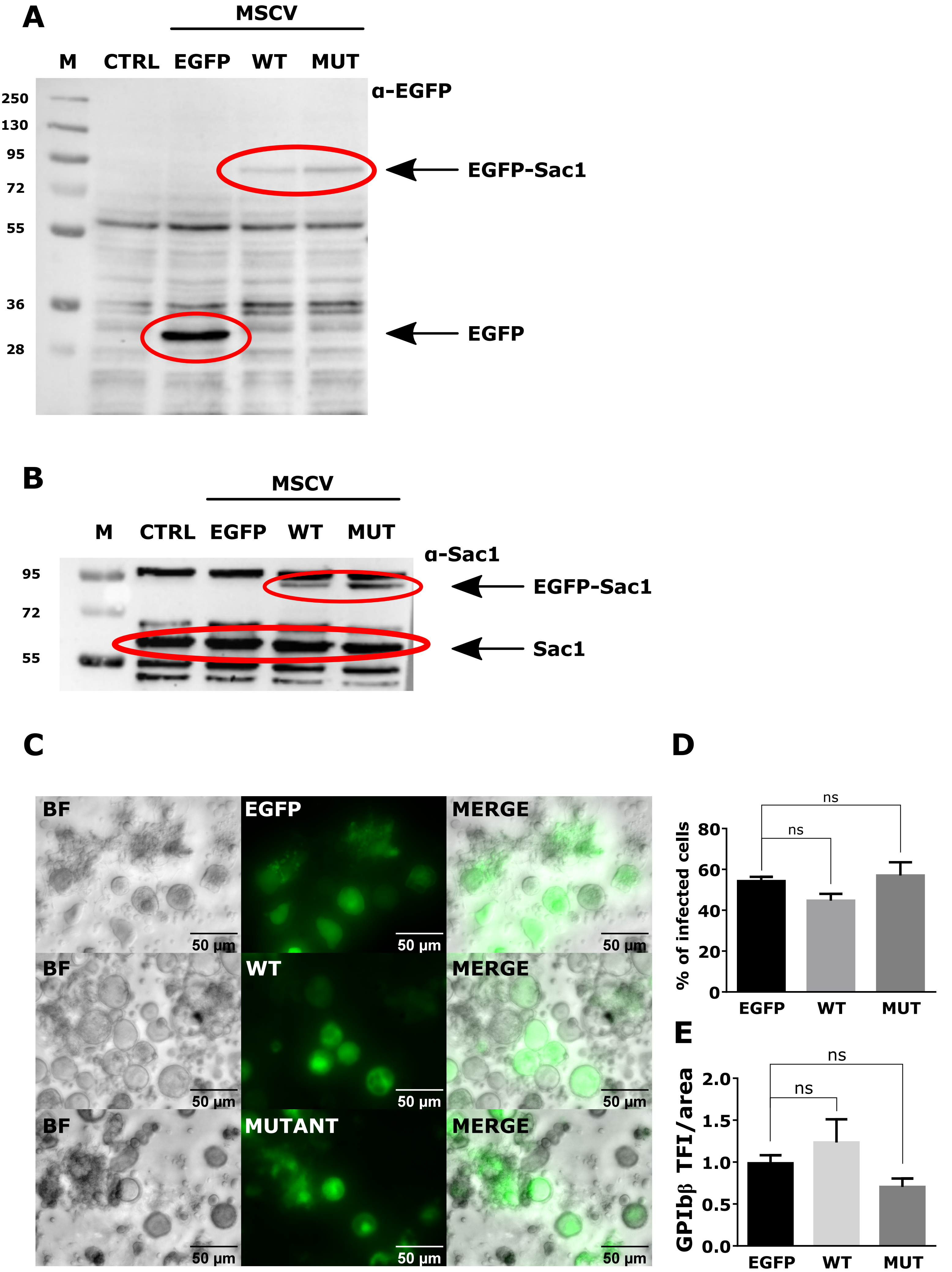

Supplemental Figure 5

A

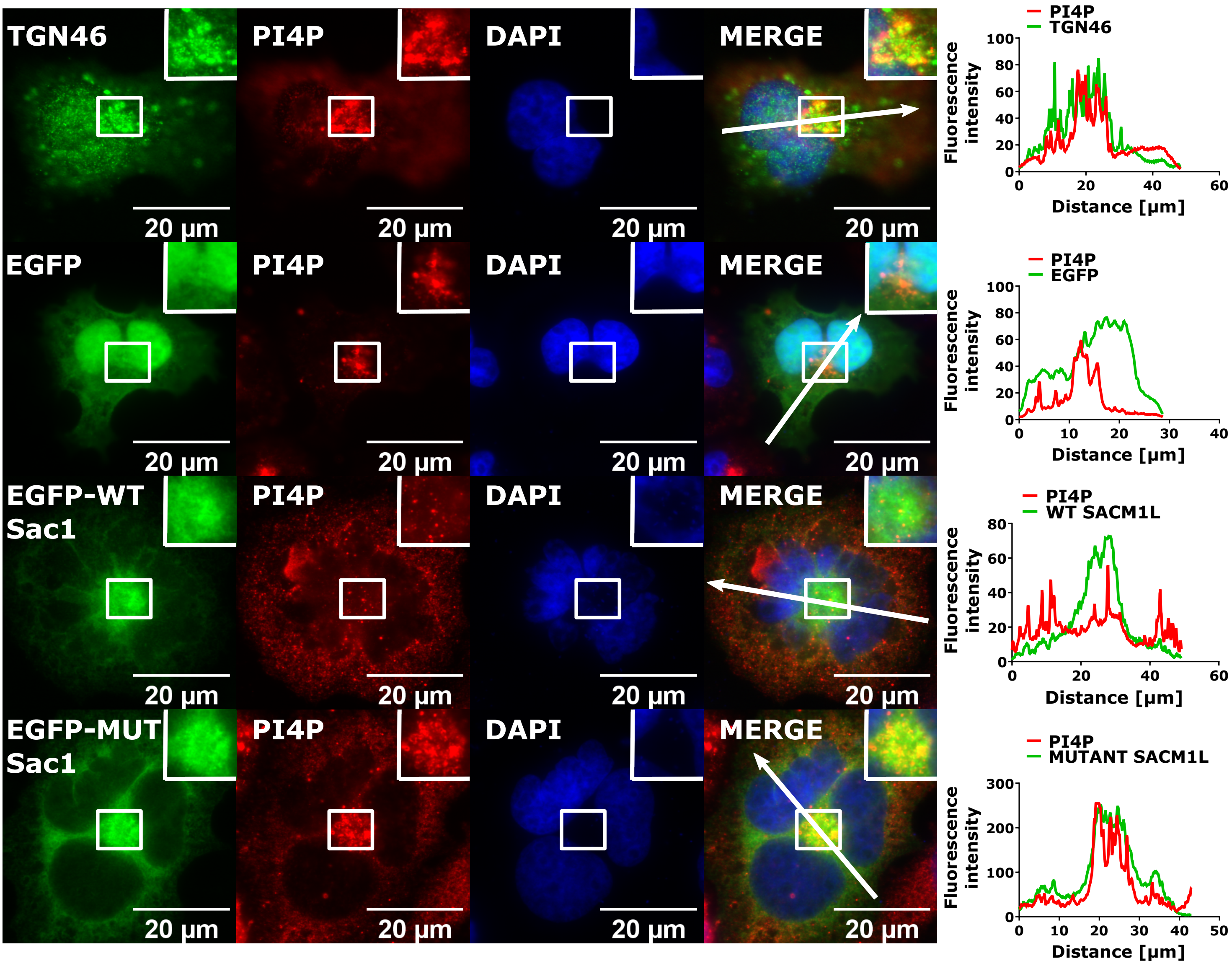

B

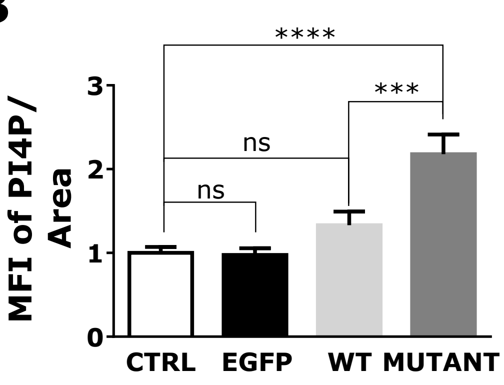

C

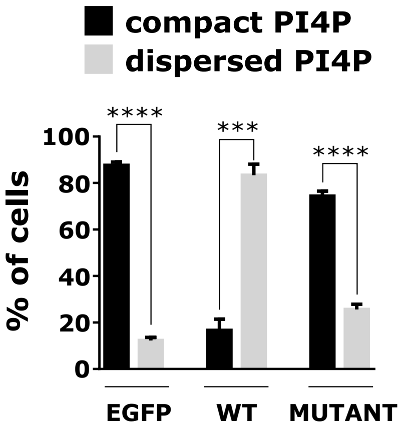

Supplemental Figure 6

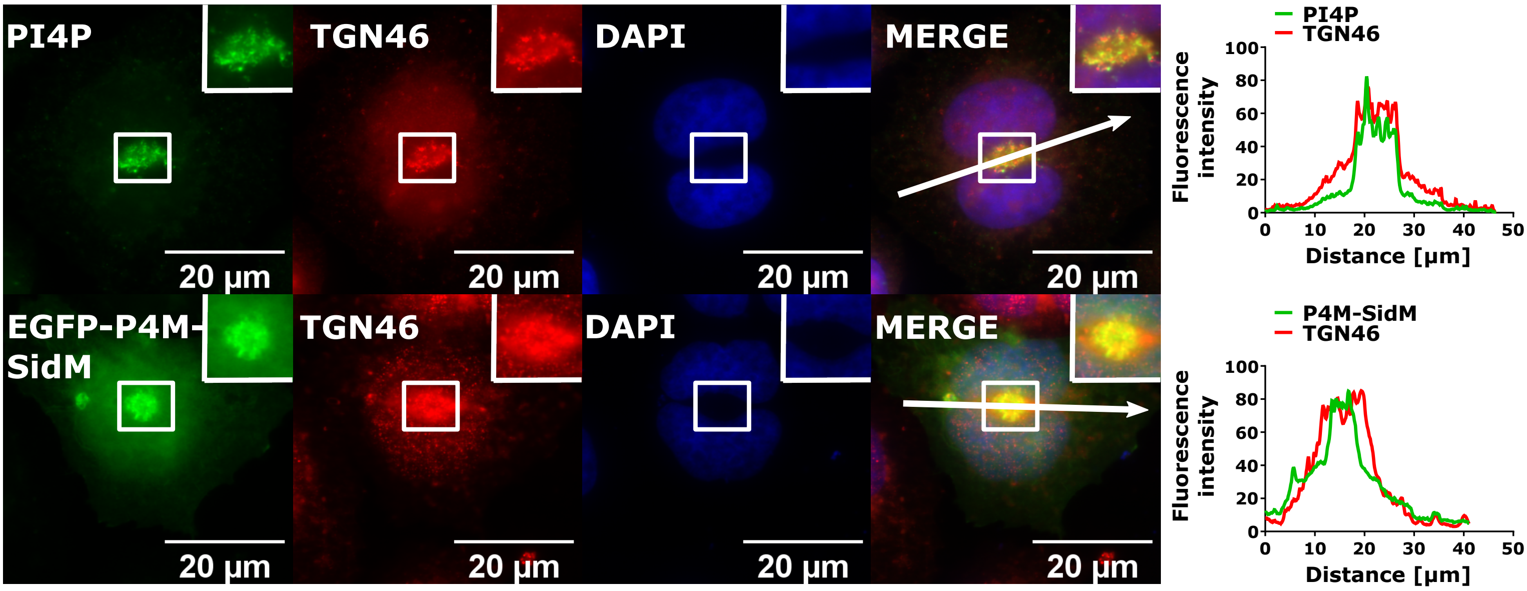

Supplemental Figure 7

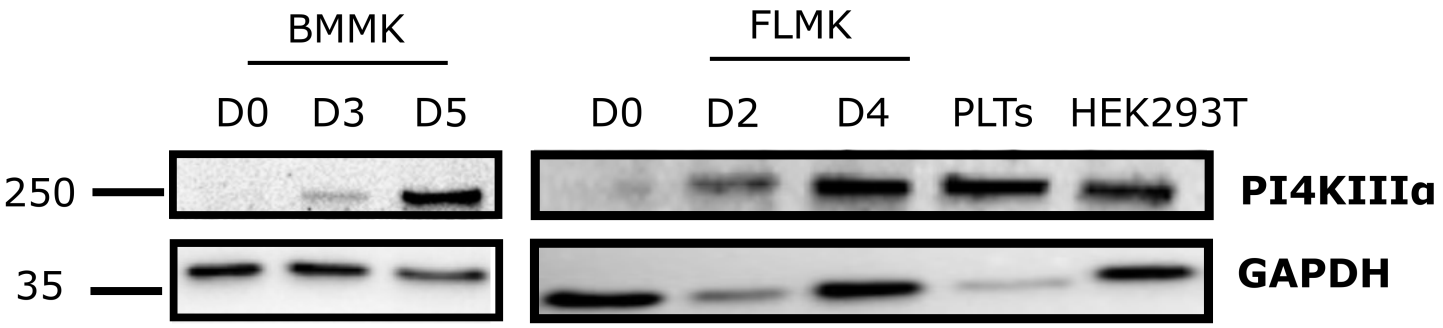

Supplement: Supplemental Figures — Supplemental Figure 1. Antibodies for Sac1 and Golgi and ER markers are specific. MKs were isolated from the mouse BM cultivated for 3 (immature) or 5 (mature) days and enriched by a BSA gradient. The cells were then fixed and stained for (A) IgG rabbit with secondary Alexa Fluor anti-rabbit 488, (B) IgG mouse with secondary Alexa Fluor anti-mouse 555 or (C) secondary antibody Alexa Fluor anti-rabbit 488. Representative images display a single confocal optical section. The scale bar of the images is 5 μm. Supplemental Figure 2. The antibody for PI4P is specific. MKs were isolated from the mouse BM cultivated for 3 (immature) or 5 (mature) days and enriched by a BSA gradient. The cells were then fixed and stained for (A) PI4P or (B) IgM mouse with secondary Alexa Fluor anti-mouse 568. Representative images display a single confocal optical section. The scale bar of the images is 5 μm. Supplemental Figure 3. The Golgi apparatus disperses during MKs maturation. MKs were isolated from (A) the mouse BM or (C) the mouse FL, cultivated for (A) 3 and 5 days or (C) 2 and 4 days, and enriched by a BSA gradient. The cells were then fixed, stained for GM130 and co-stained for TGN46. Representative images display a single confocal optical section. The scale bar of the images is 10 μm. (B and D) The number of cells with different Golgi structures was counted. At least 30 cells per condition per experiment were counted. Results in the graphs are presented as means, error bars denote ± SEM from at least 3 independent experiments. ∗, p < 0.05; ∗∗, p < 0.01; ∗∗∗, p < 0.001; ∗∗∗∗ p < 0.0001; n.s., non-significant. Supplemental Figure 4. FL-derived MKs readily express WT and mutant Sac1. (A) HEK293T cells were seeded and after 24h transfected with the indicated constructs. The cells were then lysed, separated by SDS-PAGE, blotted onto a nitrocellulose membrane, and incubated with the anti-EGFP antibody. (B) For analysis of primary MKs, mouse FL cultures were infected with EGFP-, EGFP-W [file mmc1.pdf]
